# Perceived crime and traffic safety is related to physical activity among adults in Nigeria

**DOI:** 10.1186/1471-2458-12-294

**Published:** 2012-05-17

**Authors:** Adewale L Oyeyemi, Babatunde O Adegoke, James F Sallis, Adetoyeje Y Oyeyemi, Ilse De Bourdeaudhuij

**Affiliations:** 1 Department of Physiotherapy, College of Medical Sciences, University of Maiduguri, Maiduguri, Nigeria; 2 Department of Physiotherapy, College of Medicine, University of Ibadan, Ibadan, Nigeria; 3 Department of Family and Preventive Medicine, Division of Behavioral Medicine, University of California, San Diego, California, USA; 4 Department of Movement and Sports Sciences, Faculty of Medicine and Health Sciences, Ghent University, Ghent, Belgium

**Keywords:** Walking, Transportation, Neighborhood, Africa

## Abstract

**Background:**

Neighborhood safety is inconsistently related to physical activity, but is seldom studied in developing countries. This study examined associations between perceived neighborhood safety and physical activity among Nigerian adults.

**Methods:**

In a cross-sectional study, accelerometer-based physical activity (MVPA), reported walking, perceived crime and traffic safety were measured in 219 Nigerian adults. Logistic regression analysis was conducted, and the odds ratio for meeting health guidelines for MVPA and walking was calculated in relation to four safety variables, after adjustment for potential confounders.

**Results:**

Sufficient MVPA was related to more perception of safety from traffic to walk (OR=2.28, CI=1.13- 6.25) and more safety from crime at night (OR=1.68, CI=1.07-3.64), but with less perception of safety from crime during the day to walk (OR=0.34, CI=0.06- 0.91). More crime safety during the day and night were associated with more walking.

**Conclusions:**

Perceived safety from crime and traffic were associated with physical activity among Nigerian adults. These findings provide preliminary evidence on the need to provide safe traffic and crime environments that will make it easier and more likely for African adults to be physically active.

## Background

Because of the rising prevalence of chronic non-communicable diseases occurring in developing countries [1], physical activity promotion has become an important public health agenda in sub-Saharan Africa [2]. Environmental and policy interventions have been recommended worldwide for physical activity promotion because they can influence large groups and bring about population wide change [3]. Hence, studies on neighborhood environment correlates of physical activity have increased greatly in recent years and provide evidence to guide policy interventions.

Multiple reviews have linked environmental variables like proximity to public and private recreational facilities, presence of sidewalks, and neighborhood aesthetics to recreational physical activity and walking [4-7]. Walkable neighborhoods characterized by high residential density, proximity between homes and destinations, connected street networks, and pedestrian facilities such as sidewalks have been related to walking and cycling for transportation [4-6,8]. Neighborhood safety is an important aspect of the neighborhood social environment with potential influence on physical activity behaviours.

Safety is often measured as a simple undifferentiated construct, but for physical activity two aspects of safety are particularly relevant: traffic and crime. Studies on perceived crime and traffic safety as barriers to physical activity have produced inconsistent results [9-11]. For example, in a review [6], measures of personal safety were related to walking in the expected direction in six studies, but were related in the null or unexpected direction in ten other studies. Associations between aspects of safety and physical activity have also been reported to differ between subgroups, such as men and women [12-16], African American and White adults [17], older and younger adults [18] and overweight and obese men [19]. However, many of these studies have been conducted in Western high income countries, and further investigations to understand perceived and real safety barriers for different populations, especially from developing countries, are needed. Recent findings from the developing countries of Latin America suggest that relationships between environmental attributes and physical activity in the developing countries may be complex and may be different from those in high- income developed countries [20-22]. For examples, Parra et al [20] in Curitiba, Brazil found perception of personal safety from crime when walking and bicycling, but not safety from traffic to be positively associated with physical activity in adults, however, Gomez et al [22] in Bogota, Colombia found safety from traffic to be a positive correlate of more physical activity in older adults. In another study conducted in three regions of Brazil, no significant associations were found between physical activity and traffic conditions, and safety related to walking and bicycling during the day and at night [21].

To date, only one study has been conducted on environment--physical activity relationships in Africa, and walking was found to be associated with perceived safety from crime in the expected (positive) direction [23]. However, crime safety was related only to walking and not overall physical activity, and traffic safety was related with physical activity in the unexpected (inverse) direction in the Africa study. The study was limited to university students with potential higher socioeconomic status and younger age than the general population, and the safety items utilized were not specifically tailored to the African context. Thus, the one study in Africa reflects similar discrepant findings seen in previous studies. Since perception of safety between residents of different continents is likely to vary, the use of safety items from environmental surveys constructed in Western developed countries without local adaptation is a major concern, especially in Africa where the sociocultural and physical environments are distinct.

Africa-specific studies can provide an empirical basis for relevant public health action and stimulate strategies for health promotion in Africa. The purpose of the present study was therefore to examine the association between perceived neighborhood safety variables and objectively determined and self-reported physical activity and walking among a sample of African adults in Nigeria. As a secondary aim, we also explored sex-specific associations of perceived neighborhood safety with physical activity. The hypothesis was whether findings of developed countries mostly that perception of more neighborhood safety variables is related to more physical activity would be replicated in a sample of African adults.

## Methods

### Setting and sample

Maiduguri is the largest and the capital city in Borno State, North Eastern Nigeria. The state has a population of about 4.2million people, covers an area of 72, 609sq km with a population density of 57 people/ sq km and a Gross Domestic Product (GDP) of $5.18billion [24]. Administratively, the city of Maiduguri is divided into localities, and each locality is subdivided into enumeration areas (neighborhoods) within the inner city or government reserved areas and new layout areas, with different housing patterns and street characteristics.

We recruited residents from localities classified into high and low socioeconomic status (SES) areas by the ministry of urban planning and development in Maiduguri. According to the 2003 Nigerian Demographic and Health Survey [25], high SES localities are mostly inhabited by people who are gainfully employed (elites), have more than secondary school education, and composed of many houses with functional potable water sources and modern sanitary facilities (flush toilet). Low SES localities mostly have residents who are self- employed (artisan, traders etc) or unemployed, with varying educational qualifications, and composed of few houses with functional water sources and modern sanitary facilities. Five localities each from the high and low SES areas were visited by the research team to objectively evaluate walkability characteristics using items from the Neighborhood Walkability Scale (NEWS) as a guide, and to purposively classify six neighborhoods in each locality into walkability/SES strata. For the implementation of the 1991 population census in Nigeria, a neighborhood was defined as one or more enumeration areas with a minimum of 50 households [25].

Consistent with the walkability index in previous studies [26-29], high- walkable neighborhoods in Maiduguri (localities in inner city) have a high concentration of multiple family residences, non- residential land uses (small retail stores, shops, local markets and places of worship) and streets with short block length with many alternative routes to destinations. Low- walkable neighborhoods in Maiduguri (localities in government reserved areas and new layout areas) are characterized by predominantly single family homes, few non- residential land uses for commercial purposes and streets with longer block length with fewer alternative routes to destinations. However, in practice, high-walkable neighorhoods tended to be low SES, and low-walkable neighborhoods tended to be high SES. In total, 15 high- walkable neighborhoods from low SES localities (low SES/high walkability) and 15- low walkable neighborhoods from high SES localities (high SES/low walkability) were selected for participants recruitment. This neighborhood selection method was used to identify neighborhoods of diverse walkability and SES, but unfortunately the two variables were very highly related.

Three female and two male research assistants who were indigenes of Maiduguri, and able to communicate in Hausa and English languages were trained by the investigators on accelerometer protocol, survey administration and interview and recruitment strategies. The training was done for five days within two weeks and it included observation of mock interviews to certify the interviewers prior to going in the field. The trained interviewers and the principal investigator recruited participants using a door-to-door strategy. For each selected neighborhood, households were enumerated on site and every odd-numbered household was approached for study interest and eligibility. When no one was at home or an eligible adult resided in the household but was not available, the interviewers made a maximum of five return visits for recruitment purposes. Participants who met the eligibility criteria of (1) living within the identified neighborhoods, (2) being 20 to 65years, (3) not having any disability that prevented walking, and (4) being able to complete or understand surveys in either English or Hausa languages were invited to participate in the study. Sample size was determined using a more conservative effect size (effect size statistic [d=] 0.25) than that used in a previous study [27]. We determined that 250 participants from each neighborhood stratum (high- walkable/low SES and low- walkable/high SES) were needed to detect a moderate to large effect size with more than 80% power [30].

After recruitment, two data collection points were arranged eight days apart to allow for a seven day- measurement period. At data collection one, participants were introduced to the study, their informed consent obtained, and a uniaxial accelerometer/activity monitor (CSA Model 7164; Computer Sciences Application Inc) was distributed. Participants were instructed to attach the activity monitor to an adjustable belt and to wear it firmly around the waist, positioned just above the right hip. The activity monitor was to be worn for 7 consecutive days during waking hours when the participant was not engaged in water-related activities such as bathing and swimming. For the participants who had telephone, a prompt call to remind them about wearing the accelerometers was made after the second and fourth day of data collection. At data collection two, the interviewer collected the accelerometer, measured participants height and weight using standardized procedures and conducted the survey with the participants. The interviewer followed the same call back procedures if the participant was not at home for data collection two. The average time between the end of the accelerometer measurement period and conduct of the interview if participants were not home for the scheduled data collection two appointments was two days. All participants provided informed consent, and the study was approved by Human Research Ethic Committee of the University of Maiduguri Teaching Hospital, Nigeria.

**Figure 1:**
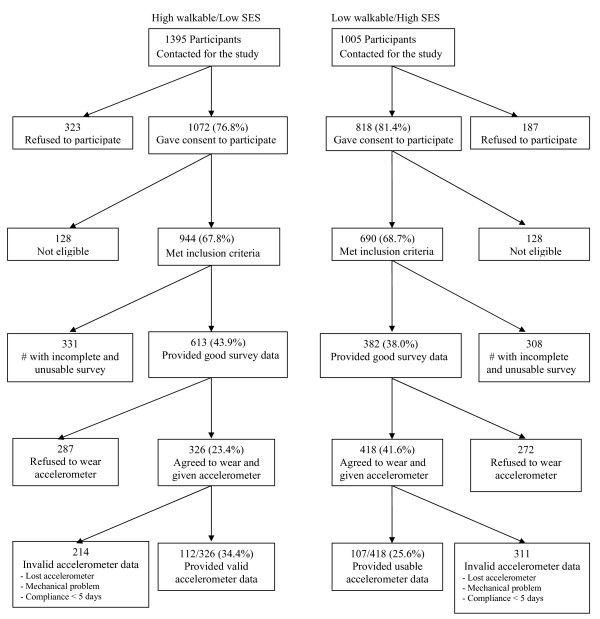
Flow Chart of Participants Recruitment

Contact was attempted with 1395 individuals (about 93/neighborhood) in the high-walkable/low SES neighborhoods and with 1005 individuals (about 67/neighborhood) in the low- walkable/high SES neighborhoods. Among the individuals contacted in the high-walkable/low SES and low- walkable/high SES neighborhoods, respectively, 323 (23.2%) and 187 (18.6%) refused participation, and 128 (9.2%) and 128 (12.7%) were not eligible. Among the individuals who agreed to participate in the high-walkable/low SES and low- walkable/high SES neighborhoods, respectively, 613 (43.9%) and 382 (38.0%) returned usable surveys. All participants who agreed to participate in the study were invited to wear accelerometers and 112/326 (34.4%) and 107/418 (25.6%) of the subset who agreed to wear accelerometers in the high-walkable/low SES and low- walkable/high SES neighborhoods respectively, provided usable objective physical activity data (Figure 1). No inducement was provided to the participants, but they were informed verbally and in the cover letter that they would be informed if their physical activity level is sufficient for health benefits after completing the study. Data were collected from August 2010 to July 2011, a period covering the rainy and dry seasons in Nigeria. The rainy season in the northern part of Nigeria lasts three to four months (JuneSeptember), with rain falling about four days in a week for as much as two hours per day during the peak period. The rest of the year is hot and dry with temperatures climbing as high as 48C (118.4F) in cities like Maiduguri.

### Measures

#### Objective physical activity

The accelerometer was used to provide an objective measure of physical activity. It collected minute-by-minute activity counts that were scored as minutes spent across the 7days in intensity levels of sedentary, light, moderate and vigorous activity based on cut-points derived from previous research [31]. An average of at least 10 hours of data for a five- day counts was required [32,33]. At least 30 minutes of continuous zero counts were defined as non wearing periods. Time spent in moderate- and vigorous-intensity activity (MVPA) was defined as accelerometer counts per minute of 1952 and higher [31,34]. The data were scored and interpreted using the MeterPlus Version 4.2 software from Santech, Inc. (http://www.meterplussoftware.com). Meeting guidelines for sufficient activity was defined according to the public health physical activity recommendation as accumulating 150min/wk or more of MVPA [35,36]. CSA accelerometers provide valid estimates of physical activity [37,38].

#### Self- reported physical activity

The short self- administered version of the International Physical Activity Questionnaire (IPAQ-SF) which included 7 items was used to assess self-reported walking. The questionnaire estimated vigorous- and moderate- intensity activities, and walking in terms of frequency (days/wk) and duration (min/day) in the last 7days. These activity categories may be treated separately by computing the total minutes of each category in a week or multiplied by their estimated values in METs and summed to gain an overall estimate of physical activity in a week (http://www.ipaq.ki.se). Total minutes of walking was the primary self-reported outcome in this study, and was dichotomized into meeting recommendations (at least 150min/wk) or not [35,36]. The test retest reliability (ICC=0.33- 0.73) and concurrent validity (=0.78- 0.92) of a Hausa version of the IPAQ-SF in Nigeria are acceptable [39]. Acceptable test retest reliability (r=0.70- 0.97) and criterion validity (r=0.23) compared with accelerometer monitoring has also been reported for IPAQ in both the developed and developing countries [40].

#### Perceived neighborhood safety factors

An adapted self- administered version of the Physical Activity Neighborhood Environment Scale (PANES) was used to assess perception of neighborhood safety factors. The first and third authors with an expert group that composed of public health scientists, geographers, town and urban planners, and housing and transportation executives adapted the PANES for use in Nigeria. The 17- item PANES was originally developed by the International Physical Activity Prevalence Study [41] and contained four indicators of crime and traffic safety that have been used internationally [18,19,42-45]. For the purpose of this study, two adapted items each were used to assess the perception of crime safety (*Walking is dangerous in my neighborhoodbecause of inadequate security from crime, molestation and harassment from hooligans, rascals and drug addicts* and *Walking is dangerous in my neighborhoodbecause of inadequate security from crime. molestation and harassment from hooligans, rascals and drug addicts*) and traffic safety (*It could be safe to bicycle in or near my neighborhood because there is little traffic* and *Walking is dangerous in my neighborhood because of the speed of traffic and aggressive driving*) in the neighborhood. Neighborhood was defined as the area within approximately one kilometer or half a mile of the participants home or any area that could be walked to in 1015 minutes. The four items were presented with the response options: strongly disagree, somewhat disagree, somewhat agree, strongly agree, dont know/ not sure, or refused to answer; and items except traffic safety for bicycling were reverse scored. For the purpose of data analysis, responses to each of the environmental variables were collapsed into categories of agree (strongly agree and somewhat agree) and disagree (strongly disagree and somewhat disagree). PANES was found to have acceptable test-retest reliability in the African population with intraclass correlation coefficients (ICC) for the four safety variables ranging from 0.43 for the item regarding crime during the day to 0.83 for the item on crime safety at night [46]. Similar test-retest findings have been reported for Swedish [47] and United States samples [44].

#### Sociodemographic characteristics

Information on age, gender, marital status, religion, income, educational level and employment status were elicited from the participants. Marital status was classified as married and not married. Educational level was classified as more than secondary school education, secondary school education, and less than secondary school education. Employment status was classified into Government/private employed, self- employed (traders, artisan etc) and unemployed (homemaker, student, retired, or unable to work). Income was categorized into 4 groupings based on NAIRA/month (15 000 NAIRA100 US Dollars).

### Statistical analyses

Descriptive statistics were computed for all variables. Chi- square statistic was used to compare physical activity and perception of neighborhood safety by gender. Separate logistic regression analyses for sufficient objective and self-reported walking as dependent variables, respectively, were conducted with the full sample to calculate the adjusted odd ratios (ORs) and 95% confidence intervals (CI) for each neighborhood safety variable. In addition, logistic regressions models were used to explore interactions between neighborhood safety variables and gender for physical activity, and variables for which there is significant interaction were separately explored for women and men. Age, gender, location (high/low SES), employment status, and educational level were the sociodemographics variables adjusted in the regression analysis. Income was not included in the regression models due to large number of missing responses (n=27) and its significant correlation with employment status (r=0.49, p<0.01) and educational level (r=0.43, p<0.01). All statistical analyses were conducted using SPSS.

## Results

### Description of sample

There were 219 participants comprising 39.3% females and 60.7% males, with mean age of 34.98.8years and BMI of 23.73.8kg/m^2^. The majority of participants were married (72.6%) and employed (72.2%). About 49% of participants had more than a secondary school education, while only 9.4% reported earning more than 90,000 Naira ( 600 US Dollars) per month. The simple majority (41.1%) of the participants were in the 2029years age group. Characteristics of the participants are shown in Table 1.

**Table 1:** Socio-demographic characteristics of the Participants (N=219)

Characteristics	n	%
**Gender**		
Women	86	39.3
Men	133	60.7
**Age Group (years)**		
2029	90	41.1
3039	78	35.6
4049	38	17.4
>50	13	5.9
Mean age ( SD)	34.98.8	
**Body Mass Index (Kgm**^**-2**^**)**		
Underweight (<18.5)	8	3.7
Normal Weight (18.5- 24.9)	136	62.1
Overweight (2529.9)	59	26.9
Obese (>30)	16	7.3
Mean BMI ( SD)	23.73.8	
**Marital Status**		
Married	159	72.6
Single	60	27.4
**Ethnic group**		
Hausa/Fulani	74	33.8
Kanui/Shuwa	49	22.4
Igbo	35	16.0
Yoruba	13	5.9
Others	48	21.9
**Religion**		
Islam	126	57.7
Christianity	93	42.5
**Educational level**		
>Secondary School	107	48.9
Secondary School	78	35.6
<Secondary School	34	15.6
**Employment status**		
Government/private employed	86	39.3
Self employed	72	32.9
Unemployed	61	27.9
Monthly Income (Naira)*		
<15,000	57	29.7
16,000- 45,000	80	41.7
46,000- 90,000	37	19.3
>90,000	18	9.4

SD- Standard Deviation

*- Do not add to 219 due to missing values; 15 000 NAIRA100 US Dollars

### Physical activity and perception of neighborhood safety

Overall, 58.4% of the participants accumulated sufficient health related MVPA according to objective measurement, whereas 51.2% reported meeting the guideline for sufficient walking according to self-reported survey. While objective MVPA was significantly higher among the men (68.4%) than women (43.0%), self-reported walking was significantly higher in women (63.3%) than in men (43.5%). Few participants perceived their neighborhood as having a high crime rate making it unsafe to go on walk during the day (24.7%), much traffic on the street making it difficult to walk (26.9%), and much traffic making it difficult to ride a bicycle (11.2%). Few participants also reported their environment as having a high crime rate making it unsafe to go on walk at night (34.2%). More female participants significantly perceived their environment to be unsafe from crime and traffic (p<0.001) than did males (Table 2).

**Table 2:** Participants Physical Activity and Perception of Neighborhood Safety, Overall and by Gender

Variables	Total			Gender		
	N (%)	**Women**n (%)	**Men**n (%)	*X*^*2*^- Statistic*	*p- values*	
**Objective MVPA**				13.87	<0.001
Sufficient	128 (58.4)	37 (43.0)	91 (68.4)		
Not Sufficient	91 (41.6)	49 (57.0)	42 (31.6)		
**Self- Reported Walking**				7.53	0.006
Sufficient	104 (51.2)	50 (63.3)	54 (43.5)		
Not Sufficient	99 (48.8)	29 (36.7)	70 (56.5)		
**Traffic Safety for Bicycling**				15.89	<0.001
Safe	190 (88.8)	66 (77.8)	127 (85.5)		
Not Safe	24 (11.2)	18 (22.2)	6 (4.5)		
**Traffic Safety for Walking**				97.53	<0.001
Safe	160 (73.1)	29 (33.7)	131 (98.5)		
Not Safe	59 (26.9)	57 (66.3)	2 (1.5)		
**Crime Safety during the Day**				73.14	<0.001
Safe	165 (75.3)	41 (47.7)	124 (93.2)		
Not Safe	54 (24.7)	45 (52.3)	9 (6.8)		
**Crime Safety at Night**				29.25	<0.001
Safe	144 (65.8)	44 (55.8)	106 (79.7)		
Not Safe	75 (34.2)	38 (44.2)	27 (20.3)		

*- The statistic is to compare men vs. women

### Perceived neighborhood safety factors associated with objective physical activity

The associations between neighborhood environment safety variables and objective MVPA are presented in Table 3. After adjusting for sociodemographics, three out of the four neighborhood safety variables were significantly associated with sufficient physical activity, but one of these associations was in the unexpected direction. Participants who perceived their neighborhood to be safe from traffic to walk were more than twice likely to engage in sufficient objective MVPA than those who perceived traffic as a problem. Similarly, participants who perceived their neighborhood as safe from crime at night were 68% more likely to engage in sufficient objective MVPA than those who perceived crime at night as a problem. However, participants who perceived their neighborhood as safe from crime during the day to walk were 66% less likely to engage in sufficient objective MVPA, and this finding was in the unexpected direction. Significant interactions regarding objective MVPA were observed between gender and safety from crime at night (OR=4.35, CI=1.09- 20.01). Specifically, perception of safety from crime at night was significantly related to more objective MVPA in men (OR=3.82, CI=1.06- 14.0) but not related to objective MVPA in women.

**Table 3:** Association between Perceived Neighborhood Safety and Sufficient Health Related Objective Physical Activity, Overall and by Gender

	Total (N=219)	Women (n=86)	Men (n=133)
	Adjusted OR (95%C.I)	Adjusted OR (95%C.I)	Adjusted OR (95%C.I)
				**Traffic Safety for bicycling**
Safe	0.87 (0.29- 2.54)	0.77 (0.20- 2.87)	0.54 (0.06- 5.42)	
Not Safe	1.00	1.00	1.00	
				**Traffic Safety for walking**
Safe	2.28 (1.13- 6.25)*			
Not Safe	1.00	---	---	
				**Crime Safety during the day**
Safe	0.34 (0.06- 0.91)*			
Not Safe	1.00	---	---	
				**Crime Safety at night**
Safe	1.68 (1.07- 3.64)*	0.63 (0.16- 2.46)	3.82 (1.08- 14.00)*	
Not Safe	1.00	1.00	1.00	

CI indicates confidence intervals; and OR, odds ratio

Adjusted for age, neighborhood location, employment status, and educational level

-- Sex-specific results were only presented for safety variables for which there were significant moderating effects with gender

* P<0.05

### Perceived neighborhood safety factors associated with self- reported walking

For sufficient self-reported walking, only two out of the four safety indicators, were statistically significantly associated with meeting the guideline. Participants who perceived their neighborhood as safe from crime during the night to walk were about 7 times more likely to engage in sufficient walking (OR=6.99, CI=2.71- 18.04). Also, participants who perceived their neighborhood as safe from crime during the day to walk were about 6 times more likely to engage in sufficient walking than those who perceived crime as a problem (OR=5.92, CI=1.38- 60.59) (Table 4). Significant interactions of self-reported walking were observed between gender and safety from crime during the day (OR=8.21, CI=1.35- 54.21), and at night (OR=2.90, CI=1.28- 29.31). While women would be more likely to walk when they perceived more safety from crime during the day (OR=10.27, CI=1.57- 1831), perception of more safety from crime during the day was not significant for men. On the other hand perception of more safety from crime at night was related with more walking in men (OR=6.97, CI=2.16- 22.50) but not in women.

**Table 4:** Association between Perceived Neighborhood Safety and Self- Reported Sufficient Health Related Walking, Overall and by Gender

	Total (N=219)	Women (n=86)	Men (n=133)
	Adjusted OR (95%C.I)	Adjusted OR (95%C.I)	Adjusted OR (95%C.I)
				**Traffic Safety for bicycling**
Safe	2.26 (0.49- 10.53)			
Not Safe	1.00	---	---	
				**Traffic Safety for walking**
Safe	1.19 (0.19- 7.12)	---	---	
Not Safe	1.00			
				**Crime Safety during the day**
Safe	5.92 (1.38- 60.59)*	10.27 (1.57- 18.31)*	3.34 (0.47-16.28)	
Not Safe	1.00	1.00	1.00	
**Crime Safety at night**				
Safe	6.99 (2.71- 18.04)*	3.09 (0.56- 17.02)	6.97 (2.16- 22.50)*	
Not Safe	1.00	1.00	1.00	

CI indicates confidence intervals; and OR, odds ratio

Adjusted for age, neighborhood location, employment status, and educational level

-- Sex-specific results were only presented for safety variables for which there were significant moderating effects with gender

* P<0.05

## Discussion

The results of this study support some relationships between neighborhood safety and physical activity among Nigerian adults. Though most variables of perceived safety from crime and traffic were associated as expected with both objective and self-reported health related physical activity, perception of traffic safety to bicycle yielded weaker and/or non- significant associations in the unexpected direction. It appears that associations between safety and physical activity in this Nigerian study were somewhat more consistent than the literature from developed countries [48]. In developing countries, the importance of the social environment, including safety from crime and from traffic as key determinants of physical activity behaviors has been emphasized in recent studies [20,21,49]. To our knowledge, no previous study, except our own [23] has documented how neighborhood safety factors relate to physical activity behaviours of adults in any African country.

Perception of the neighborhood as safe from crime during the night emerged as the strongest correlate of health related physical activity. This finding was observed for both objective MVPA and self-reported walking. Consistent with some previous studies [13-15,50], this finding suggests that an unsafe neighborhood from crime at night may have negative influence on residents' physical activity. Feeling unsafe may diminish confidence in the ability to be physically active [15] or willingness to go outdoors. This is important in low income countries of Africa where neighborhood safety may be particularly salient.

The findings for safety from crime during the day were inconsistently related to physical activity outcomes, increasing the odds of self-reported walking but decreasing the odds of objective MVPA. This pattern is similar to discrepant findings in this literature in general [9,11,20,21]. Perhaps perceived safety during the day is related to walking because much walking takes place in the neighborhood, including required walking for transportation. Perceived safety from crime during the day may be related differently to total physical activity, which also reflects occupational or leisure activities that may be done elsewhere and influenced by different factors. However, it is possible that other environmental and/or personal factors unique to the developing countries may help explain the discrepant findings on associations between safety from crime during the day and physical activity outcomes in this study. Different environmental attributes and personal factors have been associated with different physical activity outcomes among adults in the developing countries [21,51], suggesting that these relationships are complex and may differ from those in high-income countries [20]. Additional study will be needed to explain present results.

Associations in the expected direction were observed between perception of safety from traffic for walking and meeting recommendations for objective MVPA. This result suggests that heavy traffic may be a barrier to physical activity and provides preliminary evidence of the need to provide safe traffic environments to support physical activity in Africa. However, our finding may not help to resolve discrepant findings across studies because traffic safety for bicycling was not related to objective physical activity in the present study. The lack of findings for safety to bicycle is not surprising because bicycling rates have been unstudied and appear to be low among Nigerian adults compared to the developed countries. Previous studies were also unable to provide consensus on how perception of traffic was related to physical activity [5,9,23,52,53], but this variable has not been studied extensively. Perhaps improved questions about facilities that protect bicyclists and pedestrians from traffic, and specific questions about traffic speed, traffic volume, and safety of intersections would provide more informative results.

There were a few gender differences in associations of safety variables with physical activity. While safety from crime at night appeared to be a barrier for men to engage in MVPA and walking, it was not relevant for womens physical activity. Men in Africa may have to return home at night from their occupation and be active outdoors, while women may rarely go out at night because of the societal expectation on them to take care of domestic related activities. However, women in this study tended to walk more when they perceived their neighborhoods as safe from crime during the day. Perhaps women are particularly likely to walk in the neighborhood during the day, so perceived crime during the day could inhibit this relatively common behavior.

Striking differences between men and women were found in the mean ratings of safety. On all four safety items, men reported feeling significantly safer than women. The biggest difference in perceptions, 66 percentage points, was found for traffic safety for walking, and this variable was correlated with objective MVPA for women. Because traffic volume and traffic speed may be differently related to physical activity [54], it is possible that high speed of traffic is an important barrier to physical activity that is specific to women. This evidence is an empirical rationale to implement and evaluate efforts to reduce traffic speed and aggressive driving on women and men. However, no definite conclusions on this topic can be drawn yet, because the present study had a cross-sectional design and it is the first from Africa showing this effect. Still the result shows traffic control is a potential health promotion and physical activity intervention in Africa.

There were several limitations of this study. Probability sampling was used to recruit participants from diverse neighborhoods, but the relatively small sample from a single city in Nigeria may limit generalizability. Walkability was not adjusted for in this study because walkability was confounded with SES, which reflects the reality in Nigerian cities. It is important that future studies develop neighborhood walkability and/or activity- friendliness indexes unique to Nigeria based on empirical analysis. The cross-sectional design does not allow us to determine causal relationships. Prospective or quasi- experimental studies are needed to strengthen the evidence of causality in this field. Although the scoring of objective physical activity at moderate-to-vigorous levels was consistent with other studies, [32,55], different methods have been used [56], and there is not a consensus about how to compute achievement of physical activity guidelines. The present study was conducted during two distinct seasons in Nigeria with varying weather conditions that could impact physical activity behavior. Though the perceived safety variables were specific to the neighborhood, neither of the physical activity outcomes was neighborhood-specific. This mis-match introduced error, but makes the several significant findings more notable.

The findings from the gender- specific analyses should be interpreted with caution as most of the resulting estimates were not precise. Small sample size can adversely affects precision of estimates and compromise the power of a study [30]. However, the findings can be considered preliminary and indicate the need for larger and more definitive studies, especially on effect modification of gender between neighborhood safety and physical activity in the African population.

Strengths of the study included separate measures for crime safety and traffic safety, objectively measured total physical activity, and self-report walking, which is the most common physical activity. The sample was selected to represent a range of neighborhood physical and socioeconomic environments. Though there are some inconsistencies in present findings about the relation of perceived traffic and crime safety to physical activity among Nigerian adults, the preponderance of evidence indicates that both crime and traffic safety appear to be significant barriers to physical activity. Because women felt less safe on all items, it appears that women are more affected by safety concerns.

As Africa continues to develop, it is predictable that automobile travel and chronic disease will increase. The present study is the first evidence that these components of development may be inter-connected, specifically in an African country. Thus, transportation planners should consider the likely health consequences of their decisions, and health officials should become involved in transportation planning. Every country places a high priority on reducing criminal behavior, and present findings add evidence that fear of crime may be having an effect of reducing physical activity, thus increasing risk of non-communicable diseases. It is important to attempt the replication of these findings in other developing nations in Africa, and also in Latin America where findings have been inconclusive [20,21,51]. The use of objective measures of crime and traffic crash statistics (the number of traffic accidents and pedestrian deaths, petty and violent crime incidents) and further development of culturally-appropriate measures of crime and traffic safety could improve the research. Further research using suitable measures in developing countries could lead to evidence-based recommendations for creating safer communities that make people more comfortable being physically active. Improving traffic and crime safety are likely two of numerous interventions needed to reverse the epidemics of inactivity-related non-communicable diseases in Africa.

## Conclusions

We found perception of safety from crime and traffic to be associated with health related physical activity, with some sex-specific findings for the association of crime safety during the day and night. Women were more concerned about neighborhood safety than men. The findings of the present study provide preliminary evidence on the need to provide safe traffic and crime environments that will make it easier for African adults to be physically active.

## Competing interests

The authors declare that they have no conflicting interests.

## Authors contributions

ALO conceived and designed the study, prepared the data, conducted the analysis and interpretation of data, and drafted the manuscript. JFS contributed to study design, data interpretation, and writing. BOA, AYO and IDB contributed to study design and revised the manuscript for important intellectual content. All authors read and approved the final manuscript.

## Pre-publication history

The pre-publication history for this paper can be accessed here:

http://www.biomedcentral.com/1471-2458/12/294/prepub
